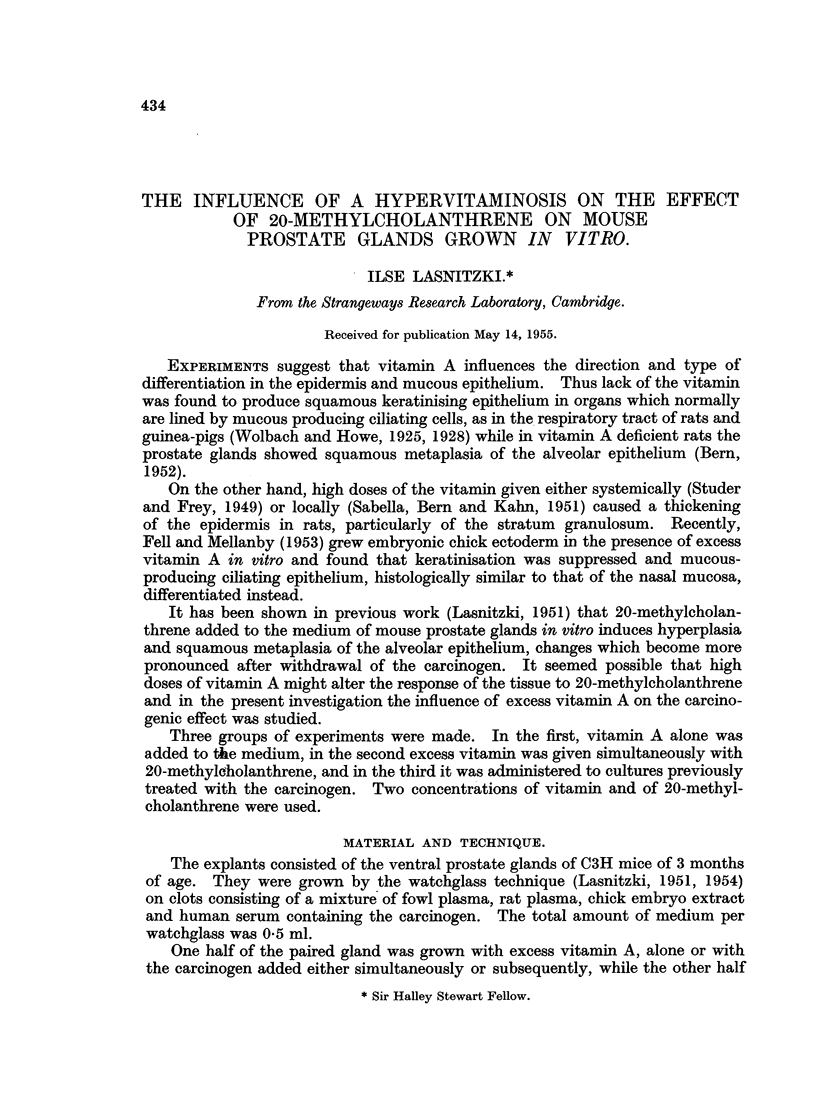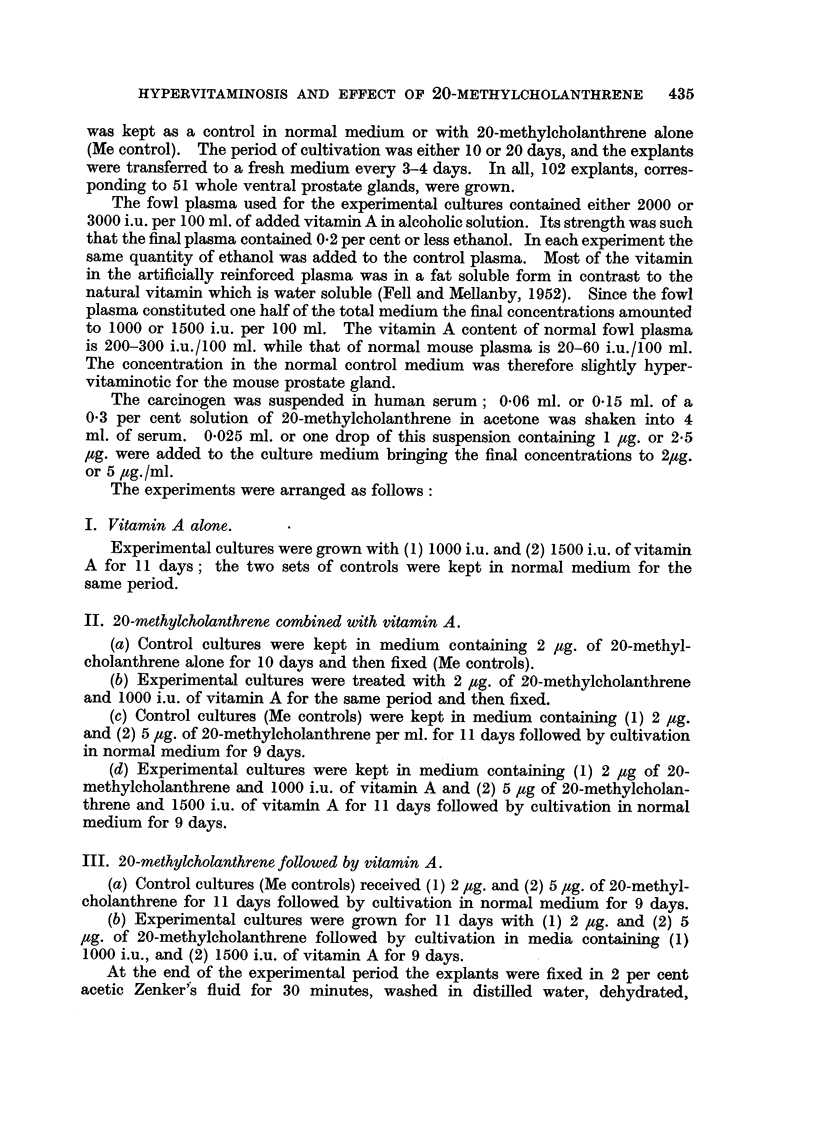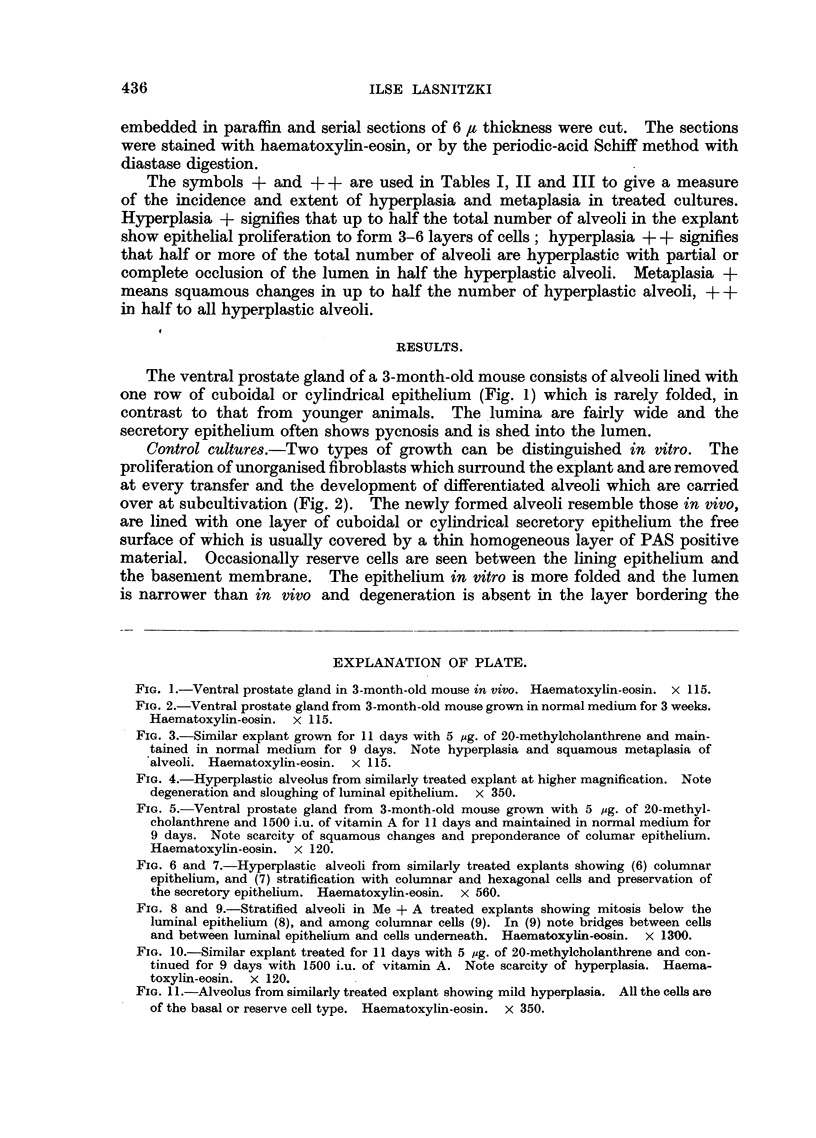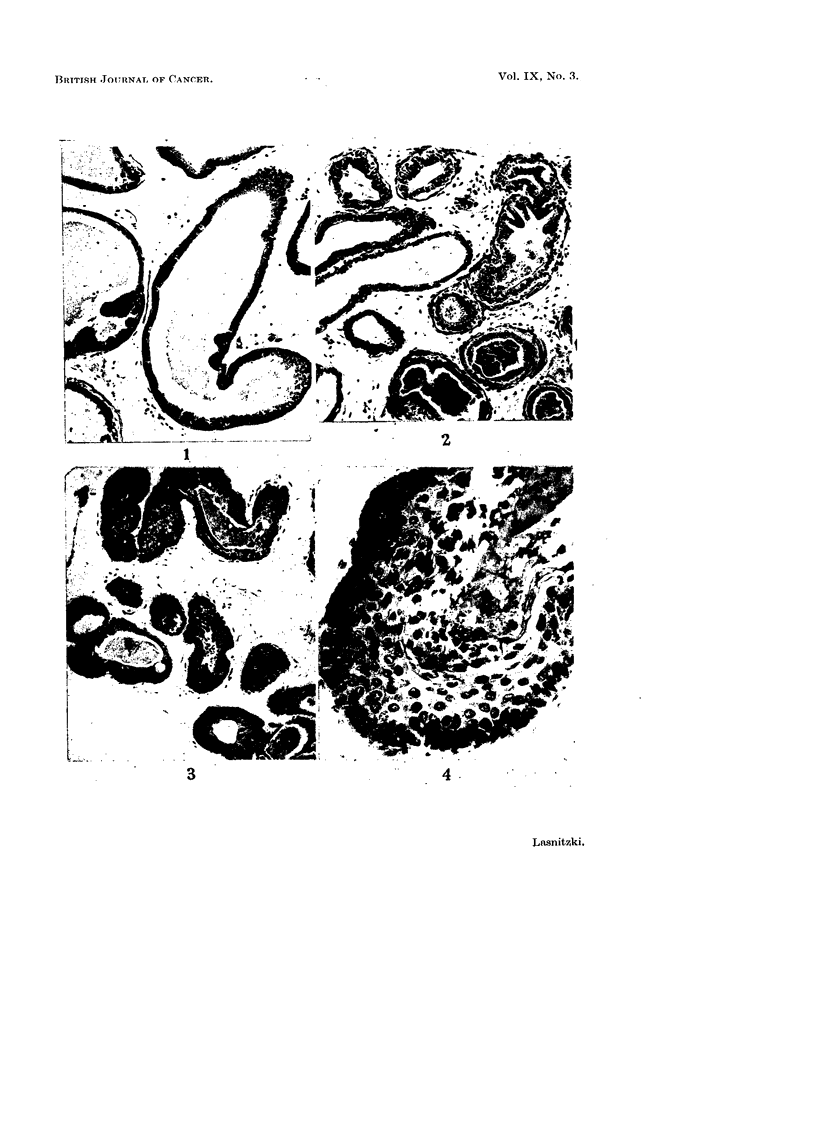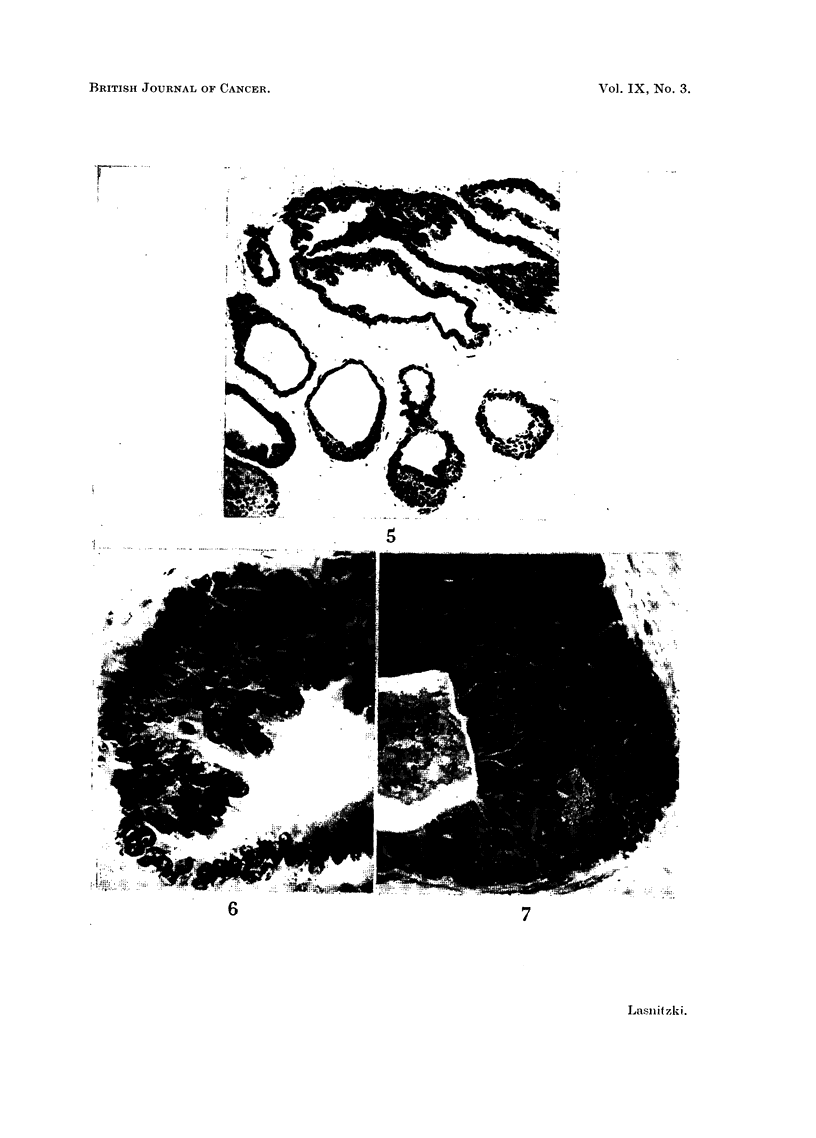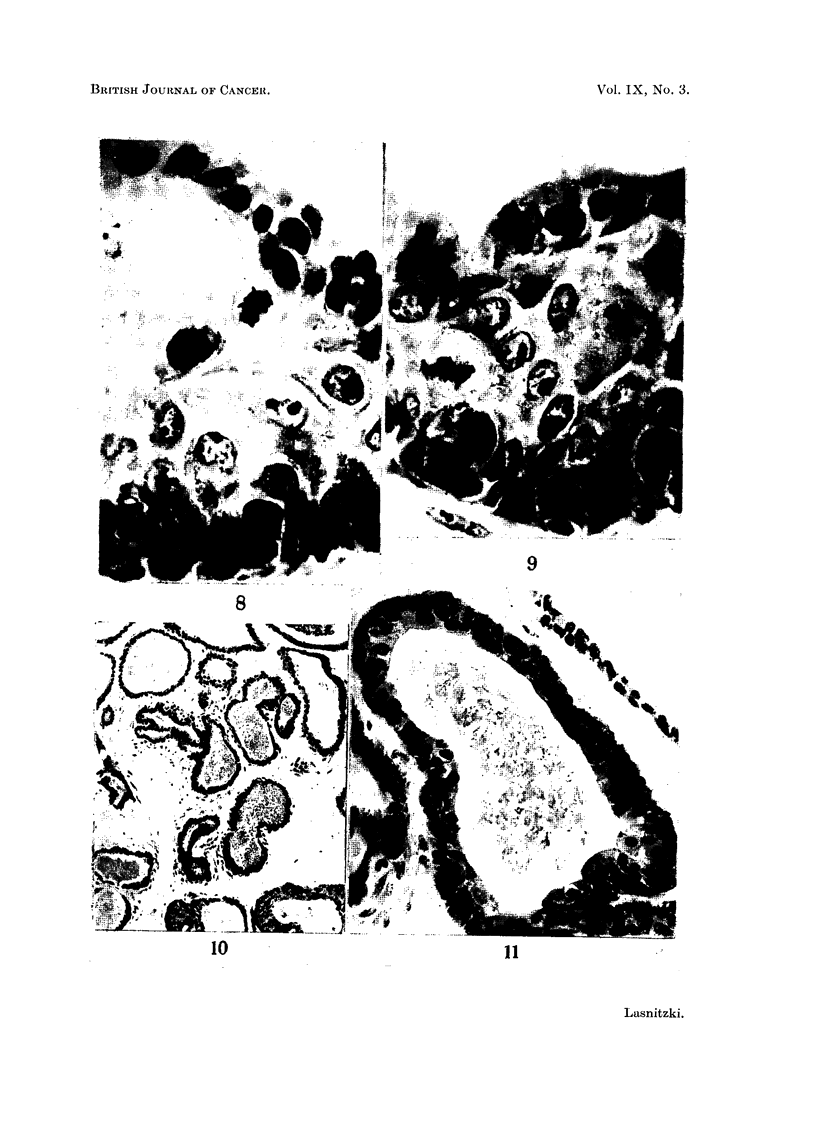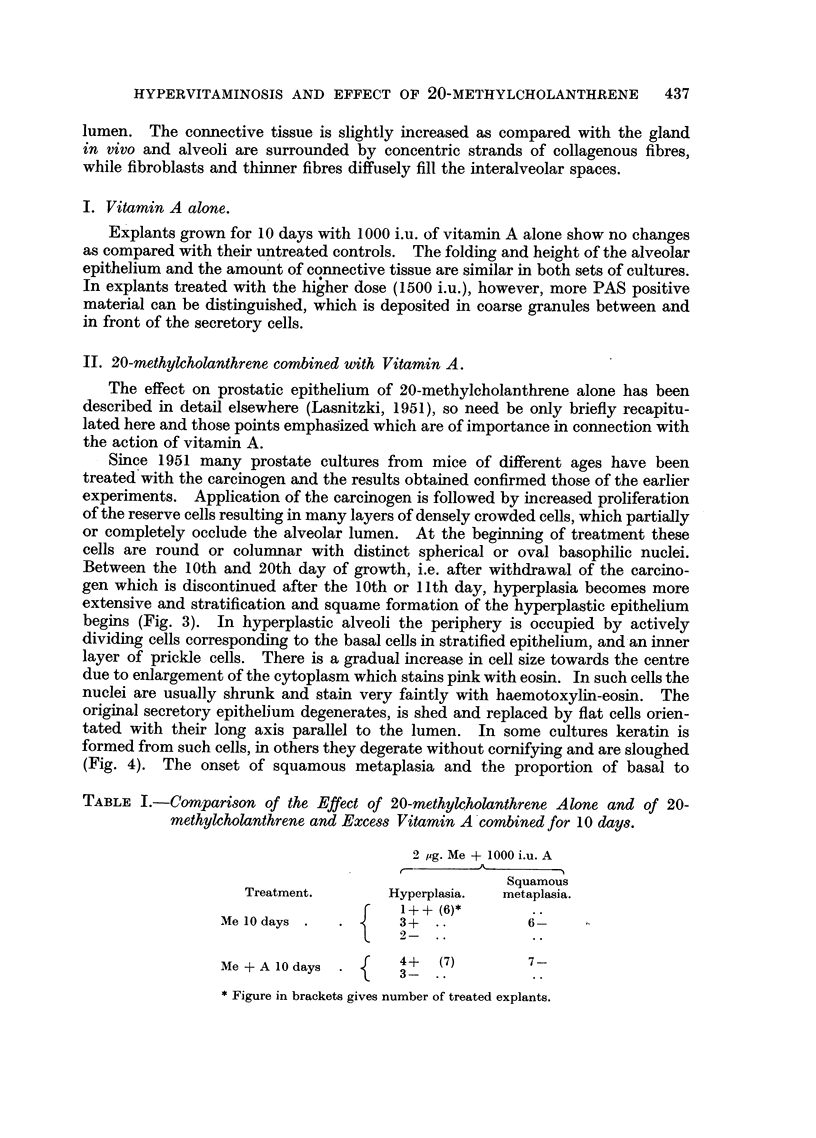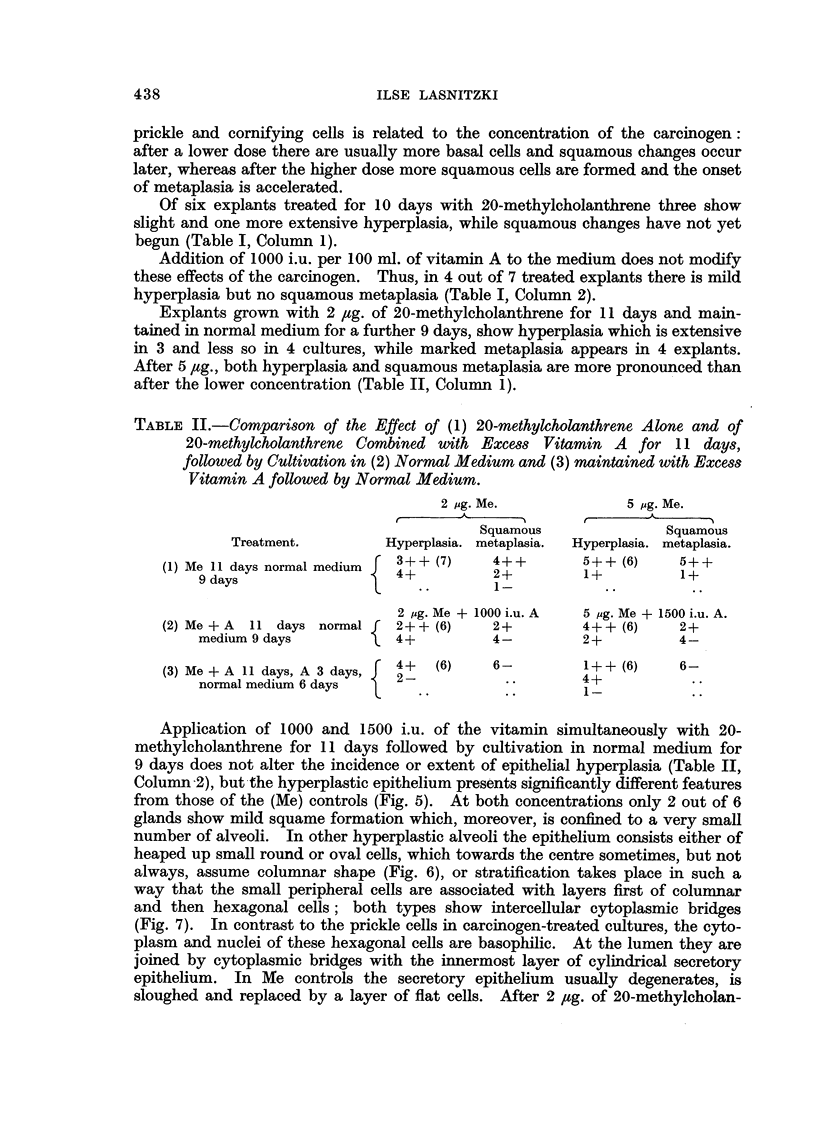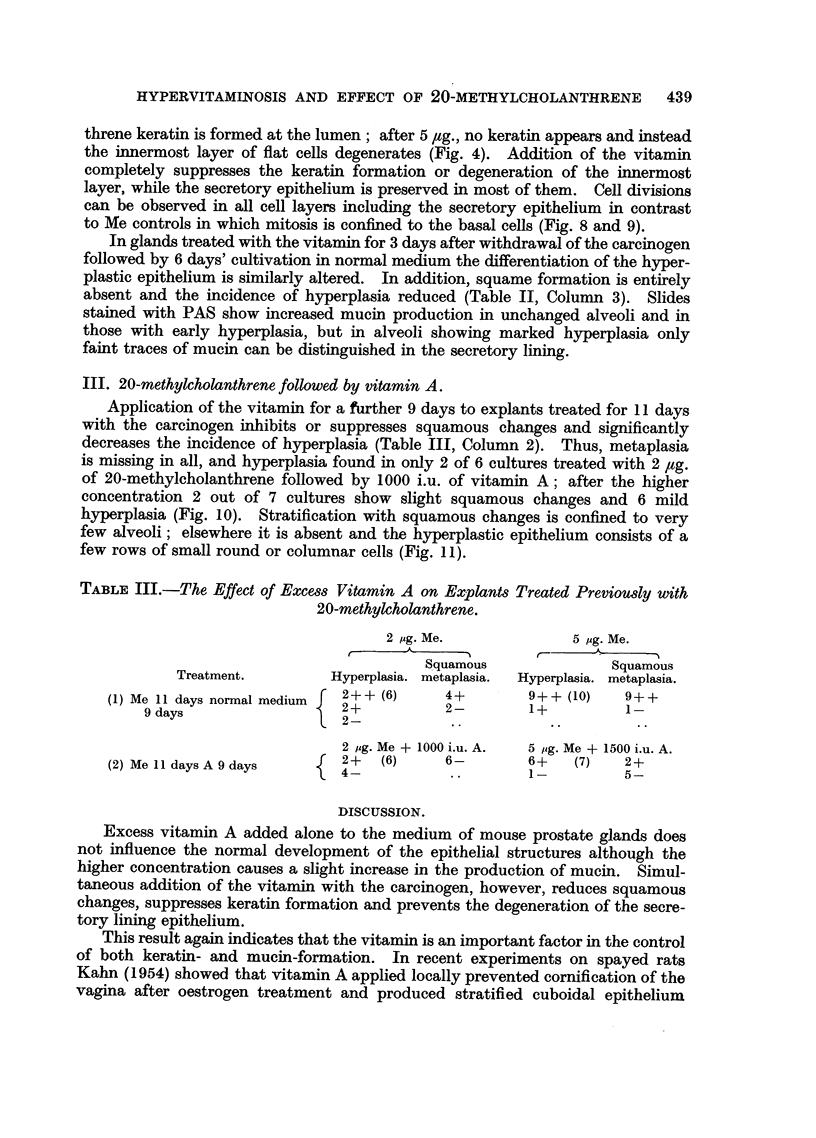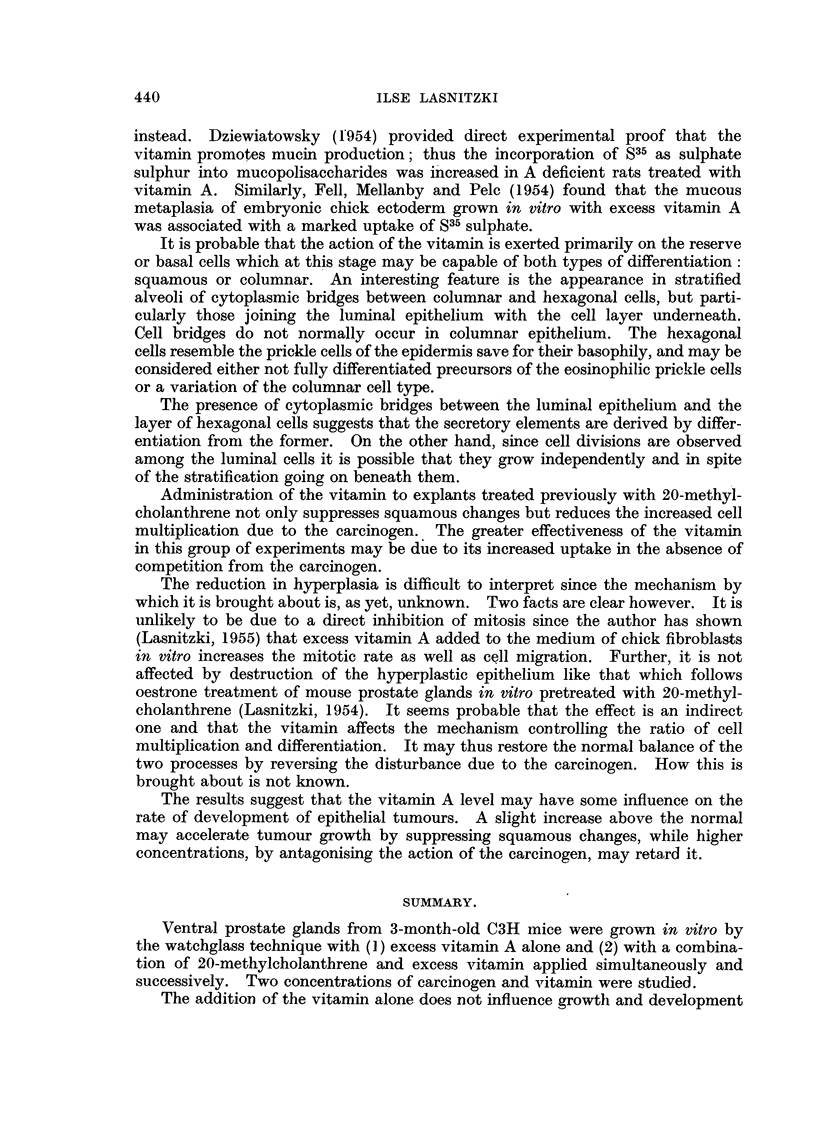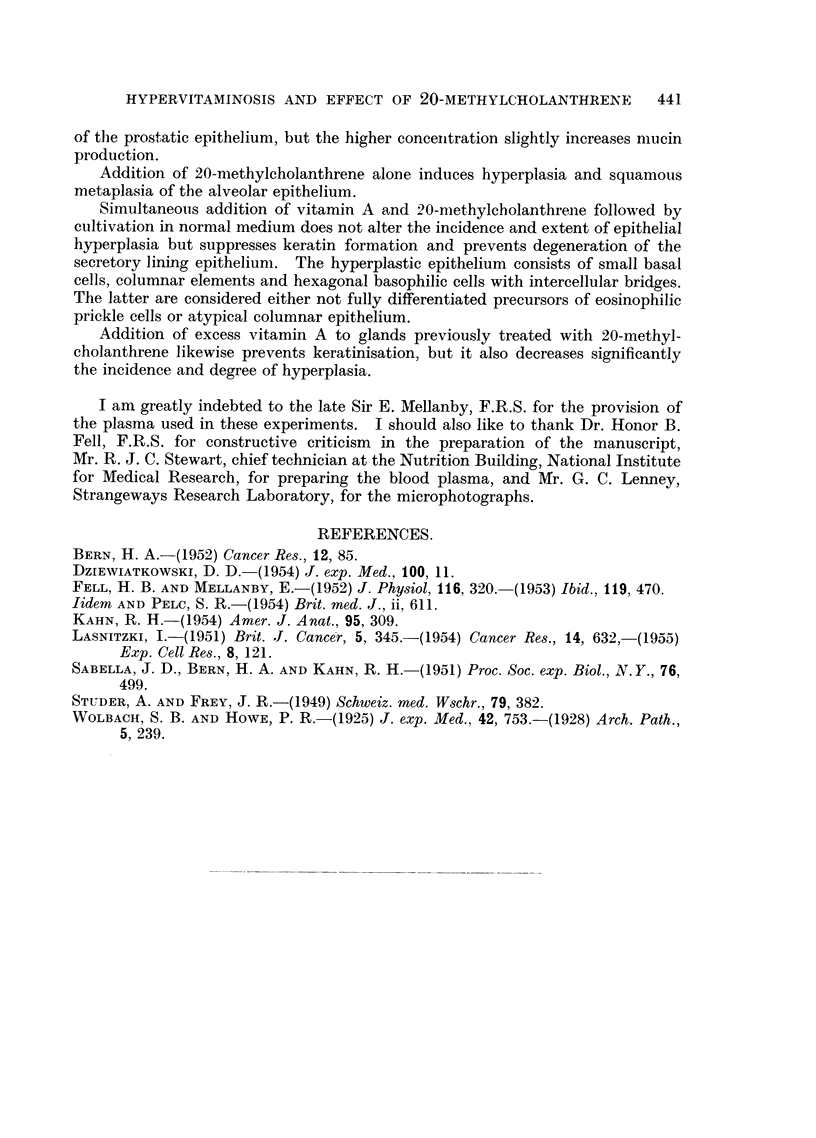# The Influence of a Hypervitaminosis on the Effect of 20-Methylcholanthrene on Mouse Prostate Glands Grown In Vitro

**DOI:** 10.1038/bjc.1955.42

**Published:** 1955-09

**Authors:** Ilse Lasnitzki

## Abstract

**Images:**


### THE INFLUENCE OF A HYPERVITAMINOSIS ON THE EFFECT OF 20-METHYLCHOLANTHRENE ON MOUSE PROSTATE GLANDS GROWN *IN VITRO.*

ILSE LASNITZKI.*

*From the Strangeways Research Laboratory, Cambridge.*

Received for publication May 14, 1955.

EXPERIMENTS suggest that vitamin A influences the direction and type of differentiation in the epidermis and mucous epithelium. Thus lack of the vitamin was found to produce squamous keratinising epithelium in organs which normally are lined by mucous producing ciliating cells, as in the respiratory tract of rats and guinea-pigs (Wolbach and Howe, 1925, 1928) while in vitamin A deficient rats the prostate glands showed squamous metaplasia of the alveolar epithelium (Bern, 1952).

On the other hand, high doses of the vitamin given either systemically (Studer and Frey, 1949) or locally (Sabella, Bern and Kahn, 1951) caused a thickening of the epidermis in rats, particularly of the stratum granulosum. Recently, Fell and Mellanby (1953) grew embryonic chick ectoderm in the presence of excess vitamin A *in vitro* and found that keratinisation was suppressed and mucous-producing ciliating epithelium, histologically similar to that of the nasal mucosa, differentiated instead.

It has been shown in previous work (Lasnitzki, 1951) that 20-methylcholanthrene added to the medium of mouse prostate glands *in vitro* induces hyperplasia and squamous metaplasia of the alveolar epithelium, changes which become more pronounced after withdrawal of the carcinogen. It seemed possible that high doses of vitamin A might alter the response of the tissue to 20-methylcholanthrene and in the present investigation the influence of excess vitamin A on the carcinogenic effect was studied.

Three groups of experiments were made. In the first, vitamin A alone was added to the medium, in the second excess vitamin was given simultaneously with 20-methylcholanthrene, and in the third it was administered to cultures previously treated with the carcinogen. Two concentrations of vitamin and of 20-methylcholanthrene were used.

### MATERIAL AND TECHNIQUE.

The explants consisted of the ventral prostate glands of C3H mice of 3 months of age. They were grown by the watchglass technique (Lasnitzki, 1951, 1954) on clots consisting of a mixture of fowl plasma, rat plasma, chick embryo extract and human serum containing the carcinogen. The total amount of medium per watchglass was 0·5 ml.

One half of the paired gland was grown with excess vitamin A, alone or with the carcinogen added either simultaneously or subsequently, while the other half

* Sir Halley Stewart Fellow.

was kept as a control in normal medium or with 20-methylcholanthrene alone (Me control). The period of cultivation was either 10 or 20 days, and the explants were transferred to a fresh medium every 3–4 days. In all, 102 explants, corresponding to 51 whole ventral prostate glands, were grown.

The fowl plasma used for the experimental cultures contained either 2000 or 3000 i.u. per 100 ml. of added vitamin A in alcoholic solution. Its strength was such that the final plasma contained 0·2 per cent or less ethanol. In each experiment the same quantity of ethanol was added to the control plasma. Most of the vitamin in the artificially reinforced plasma was in a fat soluble form in contrast to the natural vitamin which is water soluble (Fell and Mellanby, 1952). Since the fowl plasma constituted one half of the total medium the final concentrations amounted to 1000 or 1500 i.u. per 100 ml. The vitamin A content of normal fowl plasma is 200–300 i.u./100 ml. while that of normal mouse plasma is 20–60 i.u./100 ml. The concentration in the normal control medium was therefore slightly hypervitaminotic for the mouse prostate gland.

The carcinogen was suspended in human serum ; 0·06 ml. or 0·15 ml. of a 0·3 per cent solution of 20-methylcholanthrene in acetone was shaken into 4 ml. of serum. 0·025 ml. or one drop of this suspension containing 1 $\mu$g. or 2·5 $\mu$g. were added to the culture medium bringing the final concentrations to 2$\mu$g. or 5 $\mu$g./ml.

The experiments were arranged as follows :

I. *Vitamin A alone.*

Experimental cultures were grown with (1) 1000 i.u. and (2) 1500 i.u. of vitamin A for 11 days ; the two sets of controls were kept in normal medium for the same period.

II. 20-*methylcholanthrene combined with vitamin A.*

(*a*) Control cultures were kept in medium containing 2 $\mu$g. of 20-methylcholanthrene alone for 10 days and then fixed (Me controls).

(*b*) Experimental cultures were treated with 2 $\mu$g. of 20-methylcholanthrene and 1000 i.u. of vitamin A for the same period and then fixed.

(*c*) Control cultures (Me controls) were kept in medium containing (1) 2 $\mu$g. and (2) 5 $\mu$g. of 20-methylcholanthrene per ml. for 11 days followed by cultivation in normal medium for 9 days.

(*d*) Experimental cultures were kept in medium containing (1) 2 $\mu$g of 20-methylcholanthrene and 1000 i.u. of vitamin A and (2) 5 $\mu$g of 20-methylcholanthrene and 1500 i.u. of vitamin A for 11 days followed by cultivation in normal medium for 9 days.

III. 20-*methylcholanthrene followed by vitamin A.*

(*a*) Control cultures (Me controls) received (1) 2 $\mu$g. and (2) 5 $\mu$g. of 20-methylcholanthrene for 11 days followed by cultivation in normal medium for 9 days.

(*b*) Experimental cultures were grown for 11 days with (1) 2 $\mu$g. and (2) 5 $\mu$g. of 20-methylcholanthrene followed by cultivation in media containing (1) 1000 i.u., and (2) 1500 i.u. of vitamin A for 9 days.

At the end of the experimental period the explants were fixed in 2 per cent acetic Zenker's fluid for 30 minutes, washed in distilled water, dehydrated, embedded in paraffin and serial sections of 6 $\mu$ thickness were cut. The sections were stained with haematoxylin-eosin, or by the periodic-acid Schiff method with diastase digestion.

The symbols + and ++ are used in Tables I, II and III to give a measure of the incidence and extent of hyperplasia and metaplasia in treated cultures. Hyperplasia + signifies that up to half the total number of alveoli in the explant show epithelial proliferation to form 3–6 layers of cells ; hyperplasia ++ signifies that half or more of the total number of alveoli are hyperplastic with partial or complete occlusion of the lumen in half the hyperplastic alveoli. Metaplasia + means squamous changes in up to half the number of hyperplastic alveoli, ++ in half to all hyperplastic alveoli.

### RESULTS.

The ventral prostate gland of a 3-month-old mouse consists of alveoli lined with one row of cuboidal or cylindrical epithelium (Fig. 1) which is rarely folded, in contrast to that from younger animals. The lumina are fairly wide and the secretory epithelium often shows pycnosis and is shed into the lumen.

*Control cultures.*—Two types of growth can be distinguished *in vitro.* The proliferation of unorganised fibroblasts which surround the explant and are removed at every transfer and the development of differentiated alveoli which are carried over at subcultivation (Fig. 2). The newly formed alveoli resemble those *in vivo,* are lined with one layer of cuboidal or cylindrical secretory epithelium the free surface of which is usually covered by a thin homogeneous layer of PAS positive material. Occasionally reserve cells are seen between the lining epithelium and the basement membrane. The epithelium *in vitro* is more folded and the lumen is narrower than *in vivo* and degeneration is absent in the layer bordering the

---

### EXPLANATION OF PLATE.

FIG. 1.—Ventral prostate gland in 3-month-old mouse *in vivo.* Haematoxylin-eosin. × 115.

FIG. 2.—Ventral prostate gland from 3-month-old mouse grown in normal medium for 3 weeks. Haematoxylin-eosin. × 115.

FIG. 3.—Similar explant grown for 11 days with 5 $\mu$g. of 20-methylcholanthrene and maintained in normal medium for 9 days. Note hyperplasia and squamous metaplasia of alveoli. Haematoxylin-eosin. × 115.

FIG. 4.—Hyperplastic alveolus from similarly treated explant at higher magnification. Note degeneration and sloughing of luminal epithelium. × 350.

FIG. 5.—Ventral prostate gland from 3-month-old mouse grown with 5 $\mu$g. of 20-methylcholanthrene and 1500 i.u. of vitamin A for 11 days and maintained in normal medium for 9 days. Note scarcity of squamous changes and preponderance of columar epithelium. Haematoxylin-eosin. × 120.

FIG. 6 and 7.—Hyperplastic alveoli from similarly treated explants showing (6) columnar epithelium, and (7) stratification with columnar and hexagonal cells and preservation of the secretory epithelium. Haematoxylin-eosin. × 560.

FIG. 8 and 9.—Stratified alveoli in Me + A treated explants showing mitosis below the luminal epithelium (8), and among columnar cells (9). In (9) note bridges between cells and between luminal epithelium and cells underneath. Haematoxylin-eosin. × 1300.

FIG. 10.—Similar explant treated for 11 days with 5 $\mu$g. of 20-methylcholanthrene and continued for 9 days with 1500 i.u. of vitamin A. Note scarcity of hyperplasia. Haematoxylin-eosin. × 120.

FIG. 11.—Alveolus from similarly treated explant showing mild hyperplasia. All the cells are of the basal or reserve cell type. Haematoxylin-eosin. × 350.

1

2

3

4

Lasnitzki.

5

6

7

Lasnitzki.

8

9

10

11

Lasnitzki.

lumen. The connective tissue is slightly increased as compared with the gland *in vivo* and alveoli are surrounded by concentric strands of collagenous fibres, while fibroblasts and thinner fibres diffusely fill the interalveolar spaces.

I. *Vitamin A alone.*

Explants grown for 10 days with 1000 i.u. of vitamin A alone show no changes as compared with their untreated controls. The folding and height of the alveolar epithelium and the amount of connective tissue are similar in both sets of cultures. In explants treated with the higher dose (1500 i.u.), however, more PAS positive material can be distinguished, which is deposited in coarse granules between and in front of the secretory cells.

II. 20-*methylcholanthrene combined with Vitamin A.*

The effect on prostatic epithelium of 20-methylcholanthrene alone has been described in detail elsewhere (Lasnitzki, 1951), so need be only briefly recapitulated here and those points emphasized which are of importance in connection with the action of vitamin A.

Since 1951 many prostate cultures from mice of different ages have been treated with the carcinogen and the results obtained confirmed those of the earlier experiments. Application of the carcinogen is followed by increased proliferation of the reserve cells resulting in many layers of densely crowded cells, which partially or completely occlude the alveolar lumen. At the beginning of treatment these cells are round or columnar with distinct spherical or oval basophilic nuclei. Between the 10th and 20th day of growth, i.e. after withdrawal of the carcinogen which is discontinued after the 10th or 11th day, hyperplasia becomes more extensive and stratification and squame formation of the hyperplastic epithelium begins (Fig. 3). In hyperplastic alveoli the periphery is occupied by actively dividing cells corresponding to the basal cells in stratified epithelium, and an inner layer of prickle cells. There is a gradual increase in cell size towards the centre due to enlargement of the cytoplasm which stains pink with eosin. In such cells the nuclei are usually shrunk and stain very faintly with haemotoxylin-eosin. The original secretory epithelium degenerates, is shed and replaced by flat cells orientated with their long axis parallel to the lumen. In some cultures keratin is formed from such cells, in others they degerate without cornifying and are sloughed (Fig. 4). The onset of squamous metaplasia and the proportion of basal to

TABLE I.—*Comparison of the Effect of* 20-*methylcholanthrene Alone and of* 20-*methylcholanthrene and Excess Vitamin A combined for* 10 *days.*

| | 2 μg. Me + 1000 i.u. A | | |
|---|---|---|---|
| Treatment. | Hyperplasia. | | Squamous metaplasia. |
| Me 10 days | 1++ | (6)* | .. |
| | 3+ | .. | 6− |
| | 2− | .. | .. |
| Me + A 10 days | 4+ | (7) | 7− |
| | 3− | .. | .. |

* Figure in brackets gives number of treated explants.

prickle and cornifying cells is related to the concentration of the carcinogen: after a lower dose there are usually more basal cells and squamous changes occur later, whereas after the higher dose more squamous cells are formed and the onset of metaplasia is accelerated.

Of six explants treated for 10 days with 20-methylcholanthrene three show slight and one more extensive hyperplasia, while squamous changes have not yet begun (Table I, Column 1).

Addition of 1000 i.u. per 100 ml. of vitamin A to the medium does not modify these effects of the carcinogen. Thus, in 4 out of 7 treated explants there is mild hyperplasia but no squamous metaplasia (Table I, Column 2).

Explants grown with 2 μg. of 20-methylcholanthrene for 11 days and maintained in normal medium for a further 9 days, show hyperplasia which is extensive in 3 and less so in 4 cultures, while marked metaplasia appears in 4 explants. After 5 μg., both hyperplasia and squamous metaplasia are more pronounced than after the lower concentration (Table II, Column 1).

TABLE II.—*Comparison of the Effect of* (1) 20-*methylcholanthrene Alone and of* 20-*methylcholanthrene Combined with Excess Vitamin A for* 11 *days, followed by Cultivation in* (2) *Normal Medium and* (3) *maintained with Excess Vitamin A followed by Normal Medium.*

| | 2 μg. Me. | | 5 μg. Me. | |
|---|---|---|---|---|
| Treatment. | Hyperplasia. | Squamous metaplasia. | Hyperplasia. | Squamous metaplasia. |
| (1) Me 11 days normal medium 9 days | 3++ (7) | 4++ | 5++ (6) | 5++ |
| | 4+ | 2+ | 1+ | 1+ |
| | .. | 1− | .. | .. |
| | 2 μg. Me + 1000 i.u. A | | 5 μg. Me + 1500 i.u. A. | |
| (2) Me + A 11 days normal medium 9 days | 2++ (6) | 2+ | 4++ (6) | 2+ |
| | 4+ | 4− | 2+ | 4− |
| (3) Me + A 11 days, A 3 days, normal medium 6 days | 4+ (6) | 6− | 1++ (6) | 6− |
| | 2− | .. | 4+ | .. |
| | .. | .. | 1− | .. |

Application of 1000 and 1500 i.u. of the vitamin simultaneously with 20-methylcholanthrene for 11 days followed by cultivation in normal medium for 9 days does not alter the incidence or extent of epithelial hyperplasia (Table II, Column 2), but the hyperplastic epithelium presents significantly different features from those of the (Me) controls (Fig. 5). At both concentrations only 2 out of 6 glands show mild squame formation which, moreover, is confined to a very small number of alveoli. In other hyperplastic alveoli the epithelium consists either of heaped up small round or oval cells, which towards the centre sometimes, but not always, assume columnar shape (Fig. 6), or stratification takes place in such a way that the small peripheral cells are associated with layers first of columnar and then hexagonal cells; both types show intercellular cytoplasmic bridges (Fig. 7). In contrast to the prickle cells in carcinogen-treated cultures, the cytoplasm and nuclei of these hexagonal cells are basophilic. At the lumen they are joined by cytoplasmic bridges with the innermost layer of cylindrical secretory epithelium. In Me controls the secretory epithelium usually degenerates, is sloughed and replaced by a layer of flat cells. After 2 μg. of 20-methylcholanthrene keratin is formed at the lumen ; after 5 μg., no keratin appears and instead the innermost layer of flat cells degenerates (Fig. 4). Addition of the vitamin completely suppresses the keratin formation or degeneration of the innermost layer, while the secretory epithelium is preserved in most of them. Cell divisions can be observed in all cell layers including the secretory epithelium in contrast to Me controls in which mitosis is confined to the basal cells (Fig. 8 and 9).

In glands treated with the vitamin for 3 days after withdrawal of the carcinogen followed by 6 days' cultivation in normal medium the differentiation of the hyperplastic epithelium is similarly altered. In addition, squame formation is entirely absent and the incidence of hyperplasia reduced (Table II, Column 3). Slides stained with PAS show increased mucin production in unchanged alveoli and in those with early hyperplasia, but in alveoli showing marked hyperplasia only faint traces of mucin can be distinguished in the secretory lining.

III. *20-methylcholanthrene followed by vitamin A.*

Application of the vitamin for a further 9 days to explants treated for 11 days with the carcinogen inhibits or suppresses squamous changes and significantly decreases the incidence of hyperplasia (Table III, Column 2). Thus, metaplasia is missing in all, and hyperplasia found in only 2 of 6 cultures treated with 2 μg. of 20-methylcholanthrene followed by 1000 i.u. of vitamin A ; after the higher concentration 2 out of 7 cultures show slight squamous changes and 6 mild hyperplasia (Fig. 10). Stratification with squamous changes is confined to very few alveoli ; elsewhere it is absent and the hyperplastic epithelium consists of a few rows of small round or columnar cells (Fig. 11).

TABLE III.—*The Effect of Excess Vitamin A on Explants Treated Previously with 20-methylcholanthrene.*

| Treatment. | 2 μg. Me. Hyperplasia. | 2 μg. Me. Squamous metaplasia. | 5 μg. Me. Hyperplasia. | 5 μg. Me. Squamous metaplasia. |
|---|---|---|---|---|
| (1) Me 11 days normal medium 9 days | 2++ (6) | 4+ | 9++ (10) | 9++ |
| | 2+ | 2− | 1+ | 1− |
| | 2− | .. | .. | .. |
| | 2 μg. Me + 1000 i.u. A. | | 5 μg. Me + 1500 i.u. A. | |
| (2) Me 11 days A 9 days | 2+ (6) | 6− | 6+ (7) | 2+ |
| | 4− | .. | 1− | 5− |

DISCUSSION.

Excess vitamin A added alone to the medium of mouse prostate glands does not influence the normal development of the epithelial structures although the higher concentration causes a slight increase in the production of mucin. Simultaneous addition of the vitamin with the carcinogen, however, reduces squamous changes, suppresses keratin formation and prevents the degeneration of the secretory lining epithelium.

This result again indicates that the vitamin is an important factor in the control of both keratin- and mucin-formation. In recent experiments on spayed rats Kahn (1954) showed that vitamin A applied locally prevented cornification of the vagina after oestrogen treatment and produced stratified cuboidal epithelium instead. Dziewiatowsky (1954) provided direct experimental proof that the vitamin promotes mucin production; thus the incorporation of $S^{35}$ as sulphate sulphur into mucopolisaccharides was increased in A deficient rats treated with vitamin A. Similarly, Fell, Mellanby and Pelc (1954) found that the mucous metaplasia of embryonic chick ectoderm grown *in vitro* with excess vitamin A was associated with a marked uptake of $S^{35}$ sulphate.

It is probable that the action of the vitamin is exerted primarily on the reserve or basal cells which at this stage may be capable of both types of differentiation : squamous or columnar. An interesting feature is the appearance in stratified alveoli of cytoplasmic bridges between columnar and hexagonal cells, but particularly those joining the luminal epithelium with the cell layer underneath. Cell bridges do not normally occur in columnar epithelium. The hexagonal cells resemble the prickle cells of the epidermis save for their basophily, and may be considered either not fully differentiated precursors of the eosinophilic prickle cells or a variation of the columnar cell type.

The presence of cytoplasmic bridges between the luminal epithelium and the layer of hexagonal cells suggests that the secretory elements are derived by differentiation from the former. On the other hand, since cell divisions are observed among the luminal cells it is possible that they grow independently and in spite of the stratification going on beneath them.

Administration of the vitamin to explants treated previously with 20-methylcholanthrene not only suppresses squamous changes but reduces the increased cell multiplication due to the carcinogen. The greater effectiveness of the vitamin in this group of experiments may be due to its increased uptake in the absence of competition from the carcinogen.

The reduction in hyperplasia is difficult to interpret since the mechanism by which it is brought about is, as yet, unknown. Two facts are clear however. It is unlikely to be due to a direct inhibition of mitosis since the author has shown (Lasnitzki, 1955) that excess vitamin A added to the medium of chick fibroblasts *in vitro* increases the mitotic rate as well as cell migration. Further, it is not affected by destruction of the hyperplastic epithelium like that which follows oestrone treatment of mouse prostate glands *in vitro* pretreated with 20-methylcholanthrene (Lasnitzki, 1954). It seems probable that the effect is an indirect one and that the vitamin affects the mechanism controlling the ratio of cell multiplication and differentiation. It may thus restore the normal balance of the two processes by reversing the disturbance due to the carcinogen. How this is brought about is not known.

The results suggest that the vitamin A level may have some influence on the rate of development of epithelial tumours. A slight increase above the normal may accelerate tumour growth by suppressing squamous changes, while higher concentrations, by antagonising the action of the carcinogen, may retard it.

### SUMMARY.

Ventral prostate glands from 3-month-old C3H mice were grown *in vitro* by the watchglass technique with (1) excess vitamin A alone and (2) with a combination of 20-methylcholanthrene and excess vitamin applied simultaneously and successively. Two concentrations of carcinogen and vitamin were studied.

The addition of the vitamin alone does not influence growth and development of the prostatic epithelium, but the higher concentration slightly increases mucin production.

Addition of 20-methylcholanthrene alone induces hyperplasia and squamous metaplasia of the alveolar epithelium.

Simultaneous addition of vitamin A and 20-methylcholanthrene followed by cultivation in normal medium does not alter the incidence and extent of epithelial hyperplasia but suppresses keratin formation and prevents degeneration of the secretory lining epithelium. The hyperplastic epithelium consists of small basal cells, columnar elements and hexagonal basophilic cells with intercellular bridges. The latter are considered either not fully differentiated precursors of eosinophilic prickle cells or atypical columnar epithelium.

Addition of excess vitamin A to glands previously treated with 20-methylcholanthrene likewise prevents keratinisation, but it also decreases significantly the incidence and degree of hyperplasia.

I am greatly indebted to the late Sir E. Mellanby, F.R.S. for the provision of the plasma used in these experiments. I should also like to thank Dr. Honor B. Fell, F.R.S. for constructive criticism in the preparation of the manuscript, Mr. R. J. C. Stewart, chief technician at the Nutrition Building, National Institute for Medical Research, for preparing the blood plasma, and Mr. G. C. Lenney, Strangeways Research Laboratory, for the microphotographs.

### REFERENCES.

BERN, H. A.—(1952) *Cancer Res.*, **12**, 85.
DZIEWIATKOWSKI, D. D.—(1954) *J. exp. Med.*, **100**, 11.
FELL, H. B. AND MELLANBY, E.—(1952) *J. Physiol*, **116**, 320.—(1953) *Ibid.*, **119**, 470.
*Iidem* AND PELC, S. R.—(1954) *Brit. med. J.*, ii, 611.
KAHN, R. H.—(1954) *Amer. J. Anat.*, **95**, 309.
LASNITZKI, I.—(1951) *Brit. J. Cancer*, **5**, 345.—(1954) *Cancer Res.*, **14**, 632,—(1955) *Exp. Cell Res.*, **8**, 121.
SABELLA, J. D., BERN, H. A. AND KAHN, R. H.—(1951) *Proc. Soc. exp. Biol., N.Y.*, **76**, 499.
STUDER, A. AND FREY, J. R.—(1949) *Schweiz. med. Wschr.*, **79**, 382.
WOLBACH, S. B. AND HOWE, P. R.—(1925) *J. exp. Med.*, **42**, 753.—(1928) *Arch. Path.*, **5**, 239.